# Supplementary material for: Prospective evaluation of percutaneous hepatic perfusion with melphalan as a treatment for unresectable liver metastases from colorectal cancer
Source: PLoS One. 2022 Jan 13;17(1):e0261939. doi: 10.1371/journal.pone.0261939 (PMC8758076; doi:10.1371/journal.pone.0261939)
Supplement: S1 File — (PDF) [file pone.0261939.s002.pdf]

Phase II trial with melphalan for percutaneous chemosaturation (CS-PHP-Mephalan) in treating unresectable liver metastases of colorectal cancer

Version: 8.0 / 21-04-2015

NL45013.058.13

NTR4050

Principal Investigator
Dr. A.L. Vahrmeijer, MD, PhD
Leiden University Medical Center
Department of Surgical Oncology
P.O. Box 9600, 2300 RC Leiden
Phone +31 71 526 2309
Fax +31 71 526 6750
E-mail a.l.vahrmeijer@lumc.nl

Principal Investigator
Prof. C.J.H. van de Velde, MD, PhD
Leiden University Medical Center
Department of Surgical Oncology

Principal Investigator
Dr. C. Verhoef, MD, PhD
Erasmus Medical Center
Department of Surgical Oncology

Principal Investigator
Dr. A.R. van Erkel, MD, PhD
Leiden University Medical Center
Department of Radiology

Principal Investigator
Dr. M.C. Burgmans, MD, PhD
Leiden University Medical Center
Department of Radiology

S***tudy coordinator***
Drs. E.M. de Leede, MD
Leiden University Medical Center
Department of Surgical Oncology

Co-investigator
Erasmus Medical Center
Department of Radiology

Co-investigator
Erasmus Medical Center
Department of Medical Oncology

Co-investigator
Dr. H.W. Kapiteijn, MD, PhD
Leiden University Medical Center
Department of Medical Oncology

Co-investigator
Drs. J.R. van der Vorst, MD
Leiden University Medical Center
Department of Surgical Oncology

Co-investigator
Prof. A.J. Gelderblom, MD, PhD
Leiden University Medical Center
Department of Medical Oncology

Co-investigator
Bc. F.G.J. Tyl
Leiden University Medical Center
Department of

Co-investigator
Dr. H.H. Hartgrink, MD, PhD
Leiden University Medical Center
Department of Surgical Oncology

Trial pharmacist
J. Pander
Leiden University Medical Center
Department of Clinical Pharmacy
E-mail : trials@lumc.nl

Pathologist
Prof.dr. V.T.H.B.M. Smit, MD, PhD
Leiden University Medical Center
Department of Pathology

Statistician
Prof. dr. H. Putter, PhD
Leiden University Medical Center
Department of Medical Statistics

Independent physician
Dr. B.A. Bonsing, MD, PhD
Leiden University Medical Center
Department of Surgical Oncology
P.O.Box 9600, 2300 RC Leiden
Phone +31 71 526 3713
Fax +31 71 526 6744
E-mail : b.a.bonsing@lumc.nl

List of abbreviations and relevant definitions

ABR	ABR form, General Assessment and Registration form, is the application form that is required for submission to the accredited Ethics Committee (In Dutch, ABR = Algemene Beoordeling en Registratie)
AE	Adverse Event
AR	Adverse Reaction
CCMO	Central Committee on Research Involving Human Subjects; in Dutch: Centrale Commissie Mensgebonden Onderzoek
DSMB	Data Safety Monitoring Board
EMC	Erasmus Medisch Centrum, Rotterdam
EPD	Electronic Patient Dossier
EU	European Union
EudraCT	European drug regulatory affairs Clinical Trials
EZIS	CZ-EZIS: brand of the most commonly used electronic patient dossier
IC	Informed Consent
LUMC	Leids Universitair Medisch Centrum, Leiden
METC	Medical research ethics committee (MREC); in Dutch: medisch ethische toetsing commissie (METC)
RECIST	Response Evaluation Criteria in Solid Tumors
(S)AE	(Serious) Adverse Event
SUSAR	Suspected Unexpected Serious Adverse Reaction
ULN	Upper limit of normal
WHO	World Health Organization

Contents

List of abbreviations and relevant definitions 3
1 Summary 7
2 Rationale and Background 9
2.1 Rationale 9
2.2 Colorectal hepatic metastases 9
2.3 Clinical problem and isolated perfusion 9
2.4 Preliminary results 10
2.5 Potential advantages 13
2.6 Potential risks 13
2.7 Future applications 14
2.8 Summary of background 14
3 Objectives of the trial 15
3.1 Primary objective 15
3.2 Secondary Objective 15
4 Endpoints 15
4.1 Primary endpoints 15
4.2 Secondary endpoints 15
5 Patient selection criteria 16
5.1 Eligible patients 16
5.2 Inclusion criteria 16
5.3 Exclusion criteria 17
5.4 Pre-operative patient screening 17
5.5 Preoperative information 18
6 Study design 19
7 Isolated Hepatic Perfusion: procedure 19
7.1 Melphalan 19
7.2 Procedure 19
7.2.1 Pre-procedural angiography (Appendix B) 19
7.2.2 Post-angiography monitoring 20
7.2.3 Chemosaturation treatment (Appendix C) 20
7.2.4 Chemosaturation treatment schedule 22
7.2.5 In-ward preparation of the patient 23
7.2.6 Aftercare at the Intensive Care 23
7.2.7 Aftercare at the ward 24

8 Clinical evaluation, follow-up 24
8.1 Before perfusion 24
8.2 After treatment / Follow-up (please, see also the chart in table 2) 25
8.2.1 Blood test 25
8.2.2 Imaging 26
8.2.3 Pharmacokinetics to evaluate systemic melphalan exposure 26
8.2.4 Specimen Processing 27
9 Possible risks for PHP with melphalan 27
9.1 Risk percutaneous PHP 27
9.2 Melphalan toxicity 28
10 Statistical considerations 28
10.1 Statistical design 28
10.1.1 Primary endpoints 28
10.1.2 Secondary endpoints 29
10.1.3 Accrual and duration of the study 29
10.1.4 Safety monitoring 29
10.1.5 Early stopping 29
11 Patient registration procedure 29
11.1 Standard questions 30
11.2 Protocol specific questions 30
12 Forms and procedures for data collecting 30
12.1 Case report forms (CRFs) 30
12.2 Data flow 30
13 Reporting adverse events 31
13.1 Definitions 31
13.1.1 Complication 31
13.1.2 Severe complications 31
13.1.3 SAE (Serious Adverse Event) 31
13.1.4 SAR (Serious Adverse Reaction) 31
13.1.5 SUSAR (Suspected Unexpected Serious Adverse Reaction) 32
13.2 Reporting complications 32
13.3 Reporting complications 32
14 Ethical considerations 32
14.1 Declaration of Helsinki 32
14.2 Confidentiality 32
14.3 Informed consent 32

15 Trial insurance 32
Appendix A: WHO and RECIST Criteria 33
Appendix B: Pre-procedural angiography 35
Appendix C: Chemosaturation treatment 39
Appendix D: General anaesthesiology techniques 43
Appendix E: Case report forms 44
Appendix F: EORTC QLQ Questionnaires 42

1 Summary

**Title**	Phase II study on chemosaturation with percutaneous hepatic perfusion for unresectable isolated hepatic metastases of colorectal cancer using Melphalan<br><br>(Patiënten informatie brief: Geïsoleerde lever doorstroming met chemotherapie)
**Study design**	In this phase II two institution trial (LUMC and EMC), patients with unresectable isolated hepatic metastases and patients planned to undergo a two-stage hepatectomy will be included to receive percutaneous hepatic perfusion (PHP) using Melphalan.
**Rationale**	Each year in the Netherlands, approximately 11.000 patients are diagnosed with colorectal cancer. Fifty percent of these patients will eventually develop hepatic metastases. If these metastases are confined to the liver, in 25% of patients, a resection with curative intend can be performed. However, a significant amount of patients diagnosed with colorectal cancer die of the complications of the liver metastases. The only treatment option for these patients is systemic therapy, which aims to limit the disease and extend survival. Isolated liver perfusion has the advantage of controlling liver disease and decreasing treatment related symptoms and complications. This phase II trial aims to study the effectiveness and safety of the PHP treatment with Melphalan in patients with unresectable liver metastases or patients planned to undergo a two-stage hepatectomy.
**Objective**	Testing the RECIST 1.1 response after two percutaneous liver perfusions using the Delcath generation 2 system and Melphalan in patients with unresectable disease (6 week interval) or in patients planned to undergo a two-stage hepatectomy.
**Endpoints**	**Primary**<br>- Objective response rate expressed as the RECIST 1.1 criteria (Appendix A)<br>- Percentage of patients whose metastases turned into resectable ones<br>**Secondary**<br>- Safety of percutaneous liver perfusion with the Delcath 2nd generation system<br>- Overall survival, overall progression free survival and hepatic progression free survival<br>- Duration of the response and duration of stable disease in patients with unresectable livermetastases<br>- Quality of life (QoL), according to the EORTC QLQ-C30 and LMC21 questionnaires

**Sample size**	The following hypothesis will be used for this sample size calculation: we anticipate that 60% of patients will show a response (based on our data on open isolated hepatic perfusion). A response percentage of 60% and a sample size of 34 patients will result in a two-sided 95% confidence interval with a width of 34.6%.
**Inclusion criteria**	- Liver metastases of histologically confirmed primary colorectal adenocarcinoma<br>- Resection of primary tumor > 1 month before PHP and has fully recovered from surgery.<br>- Unresectable metastases confined to the liver based on CT-Thorax/abdomen and / or CT-PET imaging<br>- candidate for neoadjuvant chemotherapy as determined during the multidisciplinary meeting to down size the tumor<br>- Metastases measurable on CT-scan meeting criteria for target lesion(s) by RECIST 1.1<br>- No or prior systemic chemotherapy for colorectal adenocarcinoma<br>- Informed consent<br>- Life expectancy > 4 months<br>- Leukocytes ≥ 3.0 X 10^9/L<br>- Thrombocytes ≥ 100 X 10^9/L<br>- Creatinine clearance ≥ 60 ml/min<br>- APTT < 32.5 sec<br>- PT < 13.7 sec<br>- Aspartate aminotransferase (AST [SGOT]) and alanine aminotransferase (ALT [SGPT]) ≤ 2.5 times ULN, (≤ 5 times ULN if considered due to tumor)<br>- Serum bilirubin ≤ 1.5 times ULN<br>- Alkaline phosphatase ≤ 2.5 times ULN, (≤ 5 times ULN in case of livermetastases)
**Exclusion criteria**	- Biological age <18 and >65 years<br>- WHO performance status ≥ 2 (Appendix A)<br>- < 40% healthy liver tissue on CT<br>- Aberrant vascular anatomy or lesions, which impede PHP (e.g. aberrant right or left hepatic artery, severe atherosclerosis, vascular dissections). Embolization may be used to re-distribute liver vasculature.<br>- Prior Whipple's surgery<br>- Severe comorbidity (e.g. cardiovascular and pulmonary disease precluding general anaethesia, diabetes with nephropathy, active infections, other liver disease)<br>- Incompetent / Mentally disabled<br>- Pregnancy, inadequate contraception

2 Rationale and Background

2.1 Rationale

Each year in the Netherlands, approximately 11.000 patients are diagnosed with colorectal cancer. Fifty percent of these patients will eventually develop hepatic metastases. If these metastases are confined to the liver, in 25% of patients, a resection with curative intend can be performed. However, a significant number of patients diagnosed with colorectal cancer die of the complications of the liver metastases. The only treatment option for these patients is systemic therapy, which aims to limit the disease and extend survival. But some patients suffer from this systemic therapy and its side effects, or the disease is progressing, despite the therapy. For these patients, isolated liver perfusion may be an alternative for it has the advantage of controlling liver disease and decreasing treatment related symptoms and complications, in case of no extrahepatic disease. This phase II trial aims to study the effectiveness and safety of the PHP treatment with melphalan in patients with unresectable liver metastases or patients planned to undergo a two-stage hepatectomy.

2.2 Colorectal hepatic metastases

Malignancies of the liver are the third most important cause of cancer-related deaths in the world. This kind of cancer can be primary (origin in the liver) or secondary (metastases from another part of the body). The most common form of primary liver cancer is the hepatocellular carcinoma (HCC) and the most common form of secondary liver cancer is metastasis of colorectal carcinoma (CRCLM). About 30-50% of all patients with colorectal cancer, develop liver metastases synchronous: 14.5-25%, metachronous: 14.5-25% [1].

Patients with liver metastases of colorectal carcinoma often have a bad prognosis, and for these patients surgical resection is the only option with curative intend, despite the constant innovations in chemotherapy. The surgical treatment offers an acceptable morbidity, mortality rate and the 5-year survival is around 40-50%. Unfortunately only 20-25% of the patients with CRCLM qualify for surgery. The other patients can be treated with chemotherapy. A part of the patients treated with chemotherapy and with initially unresectable metastases, will become resectable because of the tumor response to chemotherapy.

2.3 Clinical problem and isolated perfusion

To accomplish better results for patients with unresectable metastases confined to the liver, isolated hepatic perfusion (IHP) has been developed. The principle of IHP is to isolate the liver from the systemic circulation by performing an operation. Subsequently the liver is flushed for an hour with

a very high dose of melphalan, leading to a high dose intensity, which could be toxic and lead to complications when administered systemically. After the procedure, the the liver is connected to the systemic circulation again[2] . Because this procedure is associated with considerable morbidity (20%) and mortality (7%), a new procedure was developed by Delcath in which hepatic infusion with simultaneous chemofiltration can be performed percutaneously (PHP system). Expected is that both morbidity and mortality will decrease significantly compared to the open liver procedure. In addition, this procedure can be performed several times.

2.4 Preliminary results

The PHP system with melphalan has been investigated in three completed clinical trials, with a total of 154 patients receiving PHP treatment. In Europe 55 patients have been treated up till now, of which 48 patients were treated with the generation II system. The majority of the 55 treated patients had uveal melanoma liver metastases. Four patients have been treated in the Netherlands at the NKI in Amsterdam.

Overall five treatment related deaths were reported. These deaths included a gastrointestinal bleed in a patient with a prior Whipple's procedure, hepatic failure in a patient with overwhelming (>90%) tumor burden, and gastric perforation in a patient who had melphalan reflux due to infusion during vascular spasm. The two remaining deaths were due to complications of neutropenia, which all led to protocol adjustments, of which the last was in 2007. Since 2009 there have not been treatment related deaths.

- Delcath system; generation I and generation II filter

The generation I system was implemented and used for study treatments. Any complication that occurred because of the system, was analysed and consequently the protocol was adjusted. For instance, after three cases of liver failure after the melphalan perfusion, the inclusion criteria were changed by protocol amendments. After two events of neutropenia and bone marrow failure, administering growth factors when necessary was implanted in the protocol. Also no previous Whipple's surgery can be performed, because of the result of an altered biliary anatomy. After cases of vasospasm during the procedure, nitro-glycerine was added to the protocol.

- Percutaneous hepatic perfusion

Hepatic infusion with percutaneous chemofiltration is already used in patients with hepatic metastases of ocular melanomas in 2005[3] . In 28 patients 74 procedures were performed. In this cohort of patients 3 mg/kg melphalan was determined to be a maximal safe dose. Mortality was 0%. In this study an average of 2.5 procedures per patient was performed, the mean length of hospital stay was 2.5 days. In another study [4] the anaesthetic, hemodynamic and metabolic aspects of the

treatment of 51 patients were reviewed (partly the same patients as the study in 2005). These patients received a total of 136 PHP procedures. Three procedures were complicated by complications. One procedure was aborted because of hypotension, bronchospasm, hypoxia and cyanosis immediately after induction of general anesthesia. The patient was successfully resuscitated and later underwent four PHP-procedures. Another patient developed asystole 10 minutes after an injection of protamine given in the ICU after the procedure. The patient was successfully resuscitated and later underwent two more PHP-procedures. The third patient had an anaphylactoid reaction to melphalan, hypotension and rash, after initiation of the melphalan infusion during the third PHP procedure. The patient was successfully resuscitated and stabilized. This patient did not undergo any additional PHP procedures.

During isolation of the inferior vena cava and hepatic perfusion, transient but significant hypotension ensues. The dedicated anesthesiologist is prepared for this and the patients are closely monitored. In the study by Miao and Pingpank no patient developed hemodynamic changes that warranted cardiopulmonary resuscitation and the hypotension could be managed with vasoactive drugs and additional fluid infusion.

- Systemic leakage

The generation II filter removes 97,8% of the melphalan from the blood; an increase compared with the 87,9% that was removed by the generation I filter.

- Open procedure

IHP was used for several years in a few centres in the world. In the Leiden University Medical Centre (LUMC), there is experience with this technique for over twenty years from the laboratory to the patient and over 130 patients were treated with IHP. After a phase I study with IHP, which contained a dose escalation study with melphalan[2] , a phase II study started in 1994. Evaluation of the first 66 patients of this phase II study show a reduction or even a complete remission of the metastases in over 50% of the patients. The ‘time to progression’ after IHP is 7.7 months (range 2.3-31.4) and the median survival is 28.8 months (figure 1) with a 40% 3-year survival (table 1) [5] . In case operative mortality is not included, median survival is 30.4 months with a 46% 3-year survival (table 1).

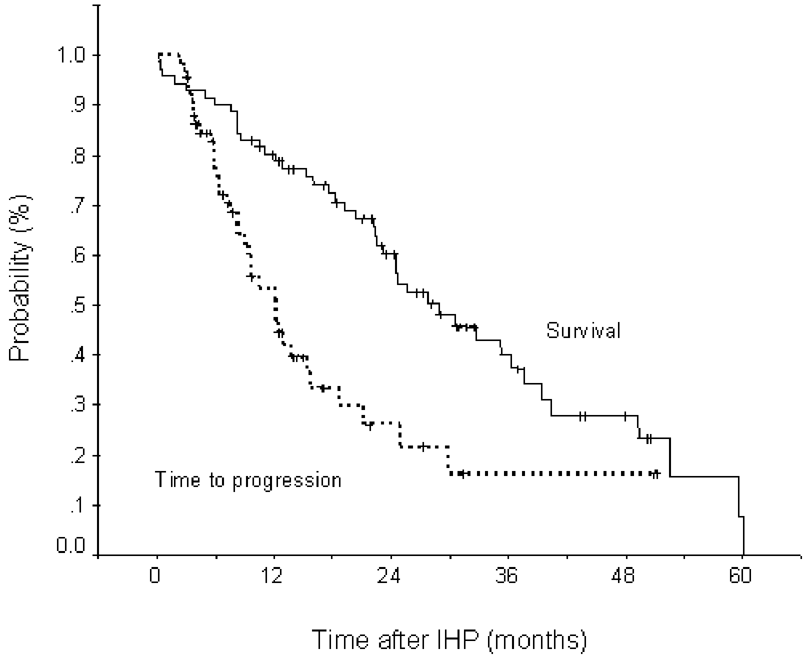

Figure 1. Kaplan-Meier curves for 'time to progression' and survival after IHP.

	Including operative mortality		*Excluding operative mortality (n=4)*	
	Median survival (months)	3-year survival (%)	Median survival (months)	3-year survival (%)
Total (n=71)	28.8	40	30.4	46
Perfusion via HA and PV (n=64)	32.7*	45	35.3**	48
No perfusion via HA (n=7)	8.6*	0	8.6**	0
No previous systemic chemotherapy (n=41)	32.7	44	36.3	50
Previous systemic chemotherapy (n=30)	24.5	34	24.5	34

Tabel 1. Survival after isolated hepatic perfusion.

* and **. Statistical significant difference between the two groups ($p<0.05$). HA= Hepatic Artery, PV= Portal Vein

A comparative case-control study compared the results of the CAIRO III trial (systemic therapy for unresectable tumors) to the results of the IHP study and showed no significant difference in survival (figure 2) [6] between the two treatments. Although no difference in survival was proven, IHP had the benefit of only having one perfusion versus systemic therapy of weeks and no systemic side effects of chemotherapeutic agents.

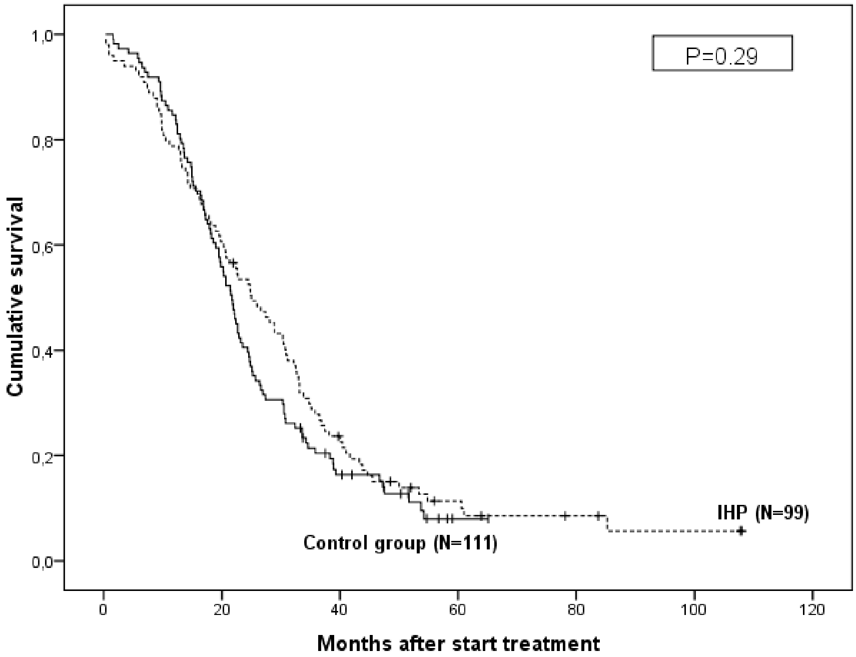

Figure 2. Case-control comparison CAIRO III and IHP

- Percutaneous Hepatic Perfusion

Data mentioned above is based on the open IHP procedure with 3.0 mg/kg melphalan, performed only once. In this percutaneous procedure is expected that both morbidity and mortality will decrease compared to the open procedure. Another aim is to increase the response rate, since the percutaneous procedure can be performed several times and a selection of patients can undergo a curative resection after tumor response to the perfusion. Based on previous results, we propose to use 2 percutaneous perfusions with melphalan at 3 mg/kg-based on ideal body weight.

2.5 Potential advantages

Percutaneous hepatic perfusion with melphalan for patients with unresectable liver metastases has the following advantages compared with the current therapy options:

- Less toxic than conventional systemic therapy for metastatic CRC
- Higher percentage of patients might qualify for radical resection after perfusion
- Minimizing length of hospital stay
- Decreasing morbidity and mortality

2.6 Potential risks

The following list of risks are possible adverse events of which the physician should be aware and the patient should be informed about.

- Hypotension; just after the filters are placed in the extracorporal circulation, there is a drop in blood pressure. Pre-procedural fluid pre-load and continuous blood pressure and ECG monitoring are needed. Vasopressors are administered to keep MAP > 65 mmHg.
- Cardiovascular safety: cardiac events did occur in previous studies; six patients experienced arrhythmias, they were all transient. Seven ischemic-type events occurred of which six cases of troponin elevation, which resolved. One patient had an acute non-T-wave myocard infarction. All cardiac events did not lead to cumulative or clinically relevant myocardial damage.
- Bleeding: there is a risk of bleeding because of the use of anticoagulation and the filter sequestration of platelets. Blood samples will be taken to monitor this and transfusion will be started as required. There is an additional risk to thrombocytopenia after the procedure, due to melphalan-induced bone marrow toxicity. Growth factor support is given when required.
- Risk of gastro-intestinal toxicity: because of a possible risk of perforation or other damage patients that had prior surgery that could affect normal hepatic biliary/vascular anatomy should be excluded (e.g. Whipple’s procedure). Hepatic artery spasm is to be treated before melphalan infusion, for instance with NG; also to prevent melphalan reflux.
- Risk of thrombocytopenia/anemia: this is closely monitored by blood sampling. It is managed with transfusion when necessary or in case of anemia by erythropoietin.

2.7 Future applications

Besides using percutaneous hepatic perfusion with melphalan for unresectable liver metastases of colorectal origin, it can also be used for liver metastases of other tumors (neuroendocrine / melanoma) and for primary liver tumors.

2.8 Summary of background

- Systemic therapy for unresectable liver metastases is not optimal due to potential systemic toxicity.
- Open isolated liver perfusion can be performed, but is associated with high morbidity and mortality. Thus, the patient can only be treated once with open IHP. The procedure cannot be repeated.
- Preliminary studies show no difference in survival between the use of systemic therapy and one

open hepatic perfusions with melphalan.

- The feasibility of percutaneous hepatic perfusions with melphalan has been tested in patients with liver metastases of ocular melanoma. The standard dose was 3 mg/kg based on ideal body weight and the average number of procedures was 3. The mean length of hospital stay was 2.5 days.
- Since percutaneous hepatic perfusions can be performed more than once, improved response is expected and more patients might qualify for a radical resection after PHP, compared with systemic treatment.

3 Objectives of the trial

3.1 Primary objective

- To determine the overall response rate of two PHP by a CT-scan in between, with a 6 week interval and 3 mg/kg melphalan in unresectable liver metastases patients.
- To determine the percentage of patients with unresectable liver metastasis that do qualify for resection after two PHP treatments.

3.2 Secondary Objective

- To assess the safety of PHP using the Generation 2nd Delcath system in patients with unresectable liver metastases from colorectal cancer.
- To determine the overall survival and overall progression free survival
- To determine the duration of response and duration of stable disease
- To determine the quality of life after two percutaneous liver perfusions.

4 Endpoints

4.1 Primary endpoints

- Response rate expressed by RECIST 1.1 criteria, after each percutaneous liver perfusion with melphalan at a six week interval
- Number of curative resections after percutaneous perfusion

4.2 Secondary endpoints

- Safety of percutaneous liver perfusion with the Delcath 2nd generation system
- Overall survival and overall progression free survival
- Duration of response and duration of stable disease

- Quality of life; measured by standardized EORTC QLQ questionnaires (see Appendix F)

5 Patient selection criteria

5.1 Eligible patients

Patients are eligible for percutaneous hepatic perfusion when the metastases in the liver are unresectable, and there is no extra hepatic disease. In a multidisciplinary meeting the patients will be discussed and decided whether the tumour is resectable or unresectable. This will also be discussed and verified with the ErasmusMC study coordinator. The following patients can be included in this trial:

- Candidate for neoadjuvant chemotherapy as determined during the multidisciplinary meeting to down size the tumor
- Patients received no, one or more courses of systemic therapy:
  - Started with the standard treatment of 5-FU/leucovorin with irinotecan or oxaliplatin, but without effect.
  - Switch of treatment without effect.
  - Not able to continue the systemic chemotherapy course because of serious side effects, suc as hand-foot syndrome
  - Progression of disease while treated with chemotherapy or within 3 months after stop.
  - No other standard systemic treatment options available

5.2 Inclusion criteria

- Informed consent
- Liver metastases of histologically confirmed primary colorectal adenocarcinoma
- Resection of primary tumor > 1 month before PHP and having fully recovered from surgery
- Unresectable metastases metastases confined to the liver based on CT-Thorax/abdomen and/or CT-PET imaging
- Metastases measurable on CT-scan
- Life expectancy > 4 months
- APTT < 32.5 sec (≤ 1.5 times ULN if considered due to tumor)
- PT < 13.7 sec (≤ 1.5 times ULN if considered due to tumor)
- Leukocytes ≥ 3.0 X 10^9/L
- Thrombocytes ≥ 100 X 10^9/L
- Creatinine Clearance ≥ 40 ml/min

- Aspartate aminotransferase (AST [SGOT]) and alanine aminotransferase (ALT [SGPT]) ≤ 2.5 times ULN (upper limit of normal). (≤ 5 times ULN if considered due to tumor)
- Serum bilirubin ≤ 1.5 times ULN
- Alkaline phosphatase ≤ 2.5 times ULN. (≤ 5 times ULN if considered due to tumor)

5.3 Exclusion criteria

- Biological age <18 and >65 years
- WHO performance status ≥ 2 (Appendix A)
- < 40% healthy liver tissue
- Aberrant vascular anatomy or vascular abnormalities (e.g. severe atherosclerosis, vascular dissections), which impede PHP
- Severe comorbidity (e.g. cardiovascular and pulmonary disease precluding general anaesthesia, diabetes with nephropathy, active infections, other liver disease)
- Whipple procedure
- Incompetent / Mentally disabled
- Pregnancy, inadequate contraception
- Intracranial lesions with a propensity to bleed (on Brain CT or MRI)

5.4 Pre-operative patient screening

- Clinical history
- Physical examination
- WHO performance status ≥ 2 (Appendix A)
- Weight
- Blood test: haematocrit, haemoglobin, thrombocytes, ALT, AST, LDH, creatinine, glucose, bilirubin, albumin, aPTT, PT, CEA
- Creatinine Clearance by eGFR
- Imaging: (within 1 month before perfusion)
    - CT-scan of thorax and abdomen including arterial phase to visualize hepatic artery anatomy
    - CT of the brain

NB 1: The aim of imaging the arterial system of the liver is to detect possible anatomic variations or vascular pathology (e.g. severe atherosclerosis, dissections) that might impede PHP.

5.5 Preoperative information

The patient is informed about the procedure in detail before consent is taken. It will be documented in EZIS (electronic patient record) what information was provided.

The patient will be informed by

- Medical oncologist; different options; systemic therapy / PHP
- Surgeon
- Radiologist who performs the PHP procedure
- Dedicated anaesthesiologist; screening and information concerning anaesthesia.

Specific issues to be addressed in the conversation between the patient and medical staff:

- Are all inclusion and exclusion criteria met?
- The general practitioner will be informed about participating in this study
- Is a proper CT, MRI and/or CT-PET-scan of the abdomen and thorax available? (within 4 weeks before the procedure)
- Renal function, risks for nephrotoxicity and the use of neurotoxic medication (see protocol*: Preventie van contrastmiddel-geïnduceerde nefropathie (CIN) bij volwassenen*)
- Coagulation status (INR, thrombocytes and use of anticoagulants)? (see protocol: s*tollingsbeleid vastgesteld voor radiologisch onderzoek en behandeling*)
- Allergies, in particular contrast-allergy (see procotol: *Contrastmiddel / Profylaxe bij ernstige contrastmiddelreactie, Radiologie*)
- Is there a medical history of cardiac disease, pulmonary disease or peptic ulcers?
- Complications: specifically mentioned should be: bleeding at inguinal insertion area, allergy, thromboembolic complications, bleeding because of gastric perforation, bleeding in general, liver failure, toxicity of melphalan.
- Handover the patient information brochures:
    - Algemene Brochure : Medisch wetenschappelijk onderzoek
    - Procedure specific brochure about percutaneous isolated hepatic perfusion
- Concomitant medications: check for chronic anticoagulant therapy, or use of ACE inhibitors, as these may need temporary adjustment for angiography as well as the perfusion procedure: (according to standard LUMC protocols)
    - Coumarines: stop (when necessary temporarily fraxiparin, stop 24h before procedure). Patient should consult ‘Trombosedienst’. Morning of procedure check INR.
    - Plavix: preferably stop 7-10 days before procedure
    - AT II inhibitors: stop 24h before perfusion

- ACE inhibitors: stop 24h before perfusion
- B-blokkers: continue

6 Study design

When all inclusion and exclusion criteria are met, a percutaneous hepatic perfusion will be performed twice, with a 6 week interval between the first and the second procedure. After each PHP treatment, the patient will be seen for a blood test and imaging (section 8), to collect data for the primary endpoint.

- If the hepatic metastases turn into a resectable size, surgery will be offered
- If there is progression of disease after the first perfusion, the second perfusion will not be performed and if possible, systemic treatment will be offered.

7 Isolated Hepatic Perfusion: procedure

7.1 Melphalan

Melphalan is an alkylating cytostatic. It binds covalently to N-7 guanine-ends in DNA and causes a quick destruction of cells. When administered systemically, myelosuppression is the dose limiting toxicity. Leukopenia and thrombocytopenia are worst at respectively day 8 and day 21. Melphalan has been used as a cytotoxic agent for isolated hepatic perfusion for over 20 years.

7.2 Procedure

Melphalan 3.0 mg/kg ideal body weight (maximal dose 220 mg) will be administered at each PHP procedure. Melphalan will be administered twice using the Delcath CS-PHP system, in combination with the Generation 2 filters, with a 6-week interval.

The CS-PHP-melphalan administration procedure consists of:

7.2.1 Pre-procedural angiography (Appendix B)

The pre-procedural angiography is done in preparation for the chemosaturation treatment. Preference is to complete this procedure at least a week prior and to PHP and to use puncture site closure device to avoid potential bleeding during PHP. During the procedure the SMA (superior mesenteric artery), coeliac trunk, common hepatic artery and possible branches of the hepatic artery to other upper abdomen organs (gastroduodenal artery, right gastric artery) will be visualized, as well as the portal vein. After delineation of the hepatic vascular anatomy, the risk of reflux of the chemotherapeutic agent to hepatico-enteric anastomoses such as the gastroduodenal or right gastric

artery is determined. If there is a possible risk of reflux during the eventual treatment, these gastro-enteric anastomoses are embolised with coils or a vascular plug. Also arterial branches distal of the site of infusion with vascular supply to the stomach, pancreas, intestines and diaphragm (i.e. aberrant right gastric artery, aberrant left hepatic artery, aberrant phrenic arteries etc) are embolised.

Preparation of the patient on the ward

The patient will be admitted in the hospital for one day; then the angiography will be made and the preoperative information mentioned above (section 5.4) will be checked and will be supplemented when necessary by the one of the investigators.

- Admission on the day of the procedure
- Check of renal function and coagulation when indicated
- Fasting for six hours for solid food and for two hours for fluids, before the procedure
- Well running peripheral venous access

7.2.2 Post-angiography monitoring

After the pre-procedural angiography the patient will be at the ward. The patient should keep bed rest for 5-8 hours. The nurse checks the inguinal insertion area, pulse, blood pressure, temperature (before discharge of when indicated), ventilation, fluid balance and whether pain management is sufficient.

Fluid balance:

- Peripheral venous access
- High oral intake because of iv contrast
- Check of urine output

Pain management:

- Additional medication can be prescribed when necessary, with approval of the attending physician
- Patient is discharged when de pain is well managed, diuresis is sufficient and no other complications occurred.

7.2.3 Chemosaturation treatment (Appendix C)

The aim of isolated hepatic perfusion is to treat patients with primary or secondary hepatic malignancies in the angio-room with chemotherapeutics. The treatment should preferably take place within one week after the pre-procedural angiography and embolization. The procedure is performed under general anaesthesia. During the procedure the liver is ‘excluded’ from the circulation by using the Delcath Hepatic CHEMOSAT Delivery System. The proper hepatic artery

is catheterized and via the Delcath Chemofuse Catheter, chemotherapeutics (melphalan hydrochloride) are eventually infused.

The Isofuse® Isolation Aspiration Catheter, inserted via the femoral vein, prevents leakage of melphalan into the systemic circulation, the latter is checked with contrast flushing. The Isofuse® catheter is a double balloon catheter. The cranial balloon shuts off the junction of the inferior vena cava and the right atrium, just above the right hepatic vein. The caudal balloon is inflated to obstruct the inferior vena cava above the renal veins, so that hepatic venous outflow is isolated from the systemic venous circulation. The chemotherapeutic agent is injected directly into the liver through the chemo delivery catheter. Chemo-containing blood from the liver, that enters the intrahepatic part of the vena cava is aspirated by the isolation aspiration catheter through the fenestrations in its wall and passed through a filtration circuit. The filtered blood is returned to the body via a catheter in the jugular vein.

This procedure enables the administration of a very high dose of chemotherapeutic agents while minimizing systemic drug exposure. This might increase the therapeutic effects without serious systemic side) effects.

7.2.4 Chemosaturation treatment schedule

1. General anaesthesia.
2. Peripheral arterial access for mean arterial pressure monitoring and pharmacokinetic sampling.
3. Placement of a sheath in the internal jugular vein for the anaesthesiologist. USE ultrasound guidance)
4. Testing of the effect of the vasopressive medication
5. Vascular access to the femoral artery (for both the sheath and the infusion catheter), the femoral vein (the sheath and Delcath double-balloon catheter) and the internal jugular vein (sheath for venous return of the filtered blood). Ultrasound guidance and single anterior wall puncture technique will be used for all venous and arterial accesses.
6. Isolation of the hepatic vein and creating a circuit outside the body, with heparin anti coagulation. Heparin is to be administered after all catheters have been placed. The ACT is supposed to be >400 during the entire procedure.
7. Placing the Delcath melphalan filters in this circuit, and adjusting the flow rates to optimize the pressure through the filters.
8. 30 minutes of CS-PHP-melphalan infusion via an angiographic injector and continuous filtration during these 30 minutes.
    - For Melphalan doses of 110 mg or less : Dilute in 250 mL of 0.9% Sodium Chloride Injection
    - For Melphalan doses of 110 mg to 220 mg : Dilute in 500 mL of 0.9% Sodium Chloride Injection
9. During these 30 minutes of melphalan infusion, every 4-6 minutes, an arteriogram is made to monitor the occurrence of vasospasm of the hepatic artery. If spasm is noted, it is treated with intra-arterial injection of 100 micrograms of nitroglycerin.
10. After the 30 min of intra-arterial perfusion is finished, hemofiltration is continued for an additional 30 minute period of washout (continuous hemofiltration lasts for a total of 60 minutes).
11. Catheters are removed after the procedure, but the access sheaths are left in until the coagulation is normalized.

12. The sheaths are removed only after coagulation is normalized with protamine, fresh frozen plasma and/or cryoprecipitate with or without transfusion of thrombocytes.

13. Patients are monitored at the ICU for the first 24 hours after the procedure.

14. After correction to safe range of possible abnormalities in coagulation, anemia and thrombocytes, patients are discharged. The following items are checked:

- INR ≤ 1.2
- Activated partial thromboplastin time (PTT) within normal range
- Hemoglobin > 9 g/dL (with or without transfusion before the procedure)
- Bicarbonate 22-28 mmol/L
- Thrombocytes > 50,000/mm^3

7.2.5 In-ward preparation of the patient

The procedure will take place while the patient is admitted in the hospital. On the day of admission patients receive paracetamol 500 mg 3 times a day. If necessary, morphinomimetics are added.

- When indicated, analgesia is continued
- Prophylactic administration of antibiotics is not indicated
- The patient is admitted the day before the procedure and the planned length of a stay is three days
- Renal function and coagulation status is checked (fasting) three hours prior to the procedure,
- Peripheral venous access is secured
- An urinary catheter is inserted
- Intravascular volume expansion should begin the night prior to the procedure with hydration.

7.2.6 Aftercare at the Intensive Care

After the procedure the patient is admitted at the Intensive Care for at least 12 hours.

- Check haemostasis site of catheter insertion
- Monitoring pulse, blood pressure, temperature, ventilation, fluid balance and pain scores
- Check coagulation (PT/INR, thrombocytes) and correction if necessary with co-factors, protamine and/or thrombocytes transfusion
- Anti-emetics if necessary, first choice: metoclopramid 2-3dd 10 mg i.v.

- Analgesia is necessary. Paracetamol 3dd 500 mg (if not sufficient, other medication can be prescribed)
- Additional medication can be prescribed when necessary, with approval of the attending physician
- On day one, blood tests are performed (haemoglobin, leukocytes number and differentiation, PT/INR, thrombocytes, AST/ALT, alkaline phosphatase, γGT, creatinine)

7.2.7 Aftercare at the ward

After 12-24 hours on the Intensive Care, the patient is transported to the normal ward, if the condition of the patient allows so. On the ward the following parameters are monitored:

- Check pulse and blood pressure three times a day
- Daily check of temperature
- Check fluid balance and pain management
- Anti-emetics if necessary, first choice: metoclopramide 2-3dd 10 mg i.v.
- Analgesia: paracetamol 3dd 500 mg (if not sufficient, other medication can be prescribed)
- Additional medication can be prescribed when necessary, with approval of the attending physician.
- After 2-3 days the patient is discharged, after a final blood test (haemoglobin, leukocytes number and differentiation, PT/INR, thrombocytes, AST/ALT, Alkaline phosphatase, γGT, creatinine).
- Colony stimulating growth factor support (neupogen/neulasta) – should be initiated upon discharge or if blood counts are followed twice a week, when ANC decreases to less than 1000

8 Clinical evaluation, follow-up

8.1 Before perfusion

- Evaluation of inclusion and exclusion criteria
- Written informed consent
- Full analysis during a multidisciplinary meeting (Medical oncologist / Surgeon / Interventional radiologist)

8.2 After treatment / Follow-up (please, see also the chart in table 2)

Follow-up moments:

- Day 9, 12, 15, 18
- After 6 weeks
- Every 3 months in the first year
- Then every 6 months.

8.2.1 Blood test

While the patient is admitted, a blood test will be performed daily and on day 9, 12, 15 and 18 (this can also be done near the patients home). Subsequently a blood test is performed weekly, until hematology, and liver functions (AST<100 U/L, ALT<112.5 U/L, bilirubin<25.5 µmol/L en GGT<175 U/L) are reduced to level I-II toxicity or are normalized.

The toxicity is scored by the 'National Cancer Institute Common Toxicity Criteria' version 4.0.

(http://www.eortc.be/services/doc/ctc/CTCAE_4.03_2010-06-14_QuickReference_5x7.pdf)

- Haematology: haemoglobin, haematocrit, thrombocytes, leukocytes differentiation, INR
- Chemistry: glucose, creatinine, sodium, potassium, bilirubin, amylase, alkaline phosphatase, ALT, AST, LDH, γ-GT, protein, albumin, bicarbonate.
- Not routinely: lactate, ammonia

8.2.2 Imaging

CT-scans (CT-thorax as well as CT-abdomen/liver) made every 3 months (after 1 year every 6 months), according a standardized protocol in the LUMC/EMC, until progression of the liver disease occurs. The first CT will be made 6-8 weeks after the first PHP treatment. Tumor response and progression will be scored expressed as WHO and RECIST 1.1 criteria (Appendix A).

	**Blood test**					**CT-scan**	**QLQ-C30**
	**Hematology**	**Chemistry**					
Point of follow-up		Standard	Lactate	Ammonia	CEA		
Screening	X	X	X	X	X	X	X
While admitted*	X	X	X	X			
At discharge	X	X					
Day 9-12-15-18 **	X	X					
6 weeks	X	X			X	X	X, X
3 months	X	X			X	X	
6 months	X	X			X	X	X
9 months	X	X			X	X	
1 year	X	X			X	X	X
1½ year	X	X			X	X	X
Etc.	X	X			X	X	

Table 2. Follow up schedule

*daily ** if blood test results are not normal by the 18th day, repeat after 1 and 2 weeks.

8.2.3 Pharmacokinetics to evaluate systemic melphalan exposure

Melphalan plasma levels will be analyzed using validated assay methods to determine systemic melphalan PK during and after melphalan percutaneous hepatic perfusion (PHP).

Blood will be collected for PK analysis during the first treatment.

For PK measurement, periphery blood will be sampled at the following times:

- Baseline
- 5 and 10 minutes after start of melphalan infusion
- Immediately upon completion of infusion (30 min.)

- 10, 20 and 30 minutes after completion of the infusion
- 5, 10, 15 and 30 minutes after completion of washout
- 1.0, 2.0, 3.5, 5.0 and 24 (+/- 2) hours after completion of washout

Approximately 5 mL of blood will be collected at each of the specified time point.

Plasma Melphalan concentrations will be determined by a validated assay and used to calculate maximum and total (area under the curve [AUC]) melphalan exposure. Samples will be stored and shipped in batches to a central laboratory designated by Delcath for analysis.

8.2.4 Blood sample Processing

1. Draw blood specimen in 6 mL sodium heparin tube.
2. Place blood collection tubes in an ice bath immediately after collection.
3. Process samples within 10 minutes of collection.

   Centrifuge samples under refrigeration; spin at 2400 rpm (1250 g) for 5 minutes at 4ºC.
4. After centrifugation, split plasma from each sodium heparin tube into 2 aliquots and store aliquots in cryovials pre-labeled with the date and time of draw, patient study identification number, cycle and day of cycle, nominal time and protocol number. Enter the same information on the PK Specimen Sheet.

   After initial collection, tubes can remain in ice bath for a maximum of 30 minutes before being frozen.
5. Store at -70ºC or colder.
6. Once frozen, do not allow samples to thaw.

9 Possible risks for PHP with melphalan

9.1 Risk percutaneous PHP

Mild complications like haematoma of the inguinal region or an aneurysma spurium subsequently to the punction of the femoral artery have to be taken into account. In rare cases a dissection of the femoral or iliac artery can occur. Although according to literature the chance is less than 2%, there is also a risk of a dissection or thrombosis of the hepatic artery. [7,8].

During the pre-procedural angiography the hepatic-enteric anastomosis will be shut off with coils,

to avoid chemotherapeutics to enter the systemic circulation. Coiling of these anastomosis is frequently used for radio-embolisation procedures. The chance of dislocation of coils during the procedure is about 2% [9]. In most of these cases a dislocated coil can be removed without further problems. Rarely, a dislocated coil can cause reduction of flow in the hepatic artery, which impedes the procedure.
During the infusion of melphalan, the intrahepatic inferior vena cava is isolated by balloon occlusion. This could lead to hypotension. Blood pressure is monitored and corrected if necessary. Non-correctable hypotension can lead to termination of the procedure.

As a result of heparinization during the hepatic perfusion, an increased risk of bleeding exists.

9.2 Melphalan toxicity

Hepatic toxicity could occur because of treatment with melphalan. Systemic toxicity (leukopenia, thrombocytopenia) could occur because of a little leakage of melphalan to the systemic circulation during PHP.

10 Statistical considerations

10.1 Statistical design

In this phase II clinical trial the primary endpoint is the response after 2 percutaneous isolated hepatic perfusions expressed by RECIST 1.1 criteria (see also section 10.2.1). In a previous clinical trial where patients were treated with 1 open hepatic perfusion, a cumulative response percentage (partial and complete) in 50 % of the patients was observed (section 2). The number of patients with a complete or partial response, according to the RECIST criteria, will be specified together with the associated confidence interval. Treatment with 2 hepatic perfusions is expected to increase the response percentage. Hypothesis: A sample size of 34 patients will yield a 95% confidence interval (two-sided) of ±16.5% around the observed proportion; assuming the observed expected proportion with response is 60% of the treated patients.

10.1.1 Primary endpoints

Response according the RECIST 1.1 criteria:

- CR (complete response) = disappearance of the lesions
- PR (partial response) = at least 30% decrease of the sum of the longest diameter of the lesions
- PD (progressive disease) = 20% increase of the sum of the longest diameter of the lesions
- SD (stable disease) = little changes that do not meet above mentioned criteria

10.1.2 Secondary endpoints

Safety of PHP with the Delcath second generation system.

- Adverse events are reported to the principal investigator
- Overall survival and overall progression free survival:

Survival analyses will be used to analyse survival. Kaplan-Meier analysis will quantify the event ‘death’ or the event ‘progression’ for the entire sample.

- Duration of response and duration of stable disease

Duration of response will be analysed as the mean amount of months, with the associated standard deviation.

- Quality of life

Quality of life will be measured by two validated questionnaires; the EORTC QLQ-C30, a general quality of life questionnaire for cancer patients and an addition, namely QLQ-LMC21 especially for patients with colorectal liver metastases. The questionnaires will be filled out by the included patients at baseline (before first perfusion), after the first perfusion and after the second perfusion. The repeated measures ANOVA will be used to analyse this data.

10.1.3 Accrual and duration of the study

The estimated accrual is up to 4 patients a month. The duration of the study will be at least 9 months, to include the 34 needed patients.

10.1.4 Safety monitoring

Adverse events will be monitored and reported directly to the principle investigator. If a severe event occurs, this will be reported to the investigator within 24 hours. Treatment related events, as well as not treatment related events will be reported.

10.1.5 Early stopping

The study coordinators reserve the right to prematurely stop the study, before the intended number of patients is included, but will only do so because of scientific of safety reasons.

If so, all participating patients will be informed within 4 weeks. Study material and CRFs will be completed as much as possible.

11 Patient registration procedure

- Possibly suitable patients are identified during the weekly multi-disciplinary hepatic conference, after that the surgeon checks the inclusion and exclusion criteria and includes the patient at the outpatient ward.

- When a patient is interested in participating, an appointment is made with a research nurse or one of the investigators. The Additional Patient Form will be provided. If the patient decides to participate, written informed consent will signed at the outpatient ward during the next visit.
- After written consent, the patient is registered at the LUMC surgical data center.

Monday – Friday   9:00 - 17:00   Tel: +31 71 526 3500

11.1 Standard questions

- Date of birth (day/month/year)
- Initials (First initial of first name, followed by first two initials of surname)
- Patient number (used in the hospital)

11.2 Protocol specific questions

- Inclusion/exclusion criteria
- Date of written informed consent
- Length and weight
- Location and number of tumors

12 Forms and procedures for data collecting

12.1 Case report forms (CRFs)

For the CRF please see Appendix E.

12.2 Data flow

- The LUMC Data Centre of the surgery department will serve as data centre for this trial. This data centre will be responsible for the registration of the patients, providing the CRFs and collecting the CRFs.
- The CRFs have to be filled out and signed by the surgeon, or an authorized staff member, as soon as the needed information is available. The responsibility for filling out the CRFs completely and correctly lies with the principal investigator.
- The CRF is designed as a booklet and can be considered a source document. After completing the booklet, the pages will be sent to the data centre. The CRF is to be kept with the patients chart. When information from the CRF cannot be entered, this must be communicated.

- To enable peer review, auditing and inspection by health care organisations, the investigator must register the identity of all participants, informed consent and copies of the CRFs. This file must be kept for 15 years after the end of the study.

13 Reporting adverse events

13.1 Definitions

13.1.1 Complication

A complication is defined as every additional medical problem occurring after treatment.

13.1.2 Severe complications

All complications matching one of the following criteria, is defined as a severe complication:

Death

Every complication that results in the death of the patient

Life-threatening

Every complication that would have resulted in the death of the patient, if no medical intervention would have had taken place

Hospitalisation

Every complication that results in a prolongation of the length of hospital stay

Readmission

Every complication that results in readmission in the hospital

Persistent disability/loss of function

Every non-life-threatening complication that results in disability or loss of function for the patient

13.1.3 SAE (Serious Adverse Event)

A SAE is defined as every severe complication, as described above, whether related to the protocol or not.

13.1.4 SAR (Serious Adverse Reaction)

A SAR is a SAE which is supposed to be a consequence of the treatment described in the study protocol.

13.1.5 SUSAR (Suspected Unexpected Serious Adverse Reaction)

A SUSAR is a severe, unexpected complication, which is considered to be a consequence of the administration of a medicament.

13.2 Reporting complications

All complications that occur within 30 days after PHP will be reported to the principal investigator.

All SAEs, SUSARs and SARs will be reported to one of the members of the protocol committee within 24 hours.

NB: All SAEs which are the result of the illness being studied, will be registered, but do not have to be reported within 24 hours.

13.3 Reporting complications

A SAE/SAR/SUSAR will be reported to the CME within 30 days.

14 Ethical considerations

14.1 Declaration of Helsinki

The investigators hereby declare that this study will be executed in accordance with the principles of the ‘Declaration of Helsinki’.

14.2 Confidentiality

Medical information of an individual patient, obtained in or through this study, is considered confidential and publishing to other parties than mentioned here is strictly prohibited.

To further ensure the confidentiality, patient information will be linked to an identification code.

Medical information can be provided to the treating physician or other medical personnel responsible for the patients’ health. Data of the study will be available on request for participating investigators, data centre and the ethical committee.

14.3 Informed consent

It’s the physicians’ responsibility to obtain a written informed consent from every participating patient, after explaining the goal, methods, possible benefits and complications of the trial.

15 Trial insurance

This study will be performed in the EMC as well as in the LUMC. The trial insurance of the LUMC and the EMC can be used during this trial.

Appendix A: WHO and RECIST Criteria

Characteristic	*WHO criteria*	*RECIST 1.1 criteria*
Measurability of lesions at baseline	1. Measurable, bidimensional (product of LD and greatest perpendicular diameter)	1. Measurable, unidimensional (LD only, size with conventional techniques 20 mm; spiral computed tomography 10 mm)
	2. Nonmeasurable/evaluable (e.g., lymphangitic pulmonary metastases, abdominal masses)	2. Nonmeasurable: all other lesions, including small lesions. Evaluable is not recommended.
Objective response	1. Measurable disease (change in sum of products of LDs and greatest perpendicular diameters, no maximum number of lesions specified)	1. Target lesions (change in sum of LDs, maximum of 5 per organ up to 10 total [more than one organ])
	CR: disappearance of all known disease, confirmed at 4 wk	CR: disappearance of all target lesions, confirmed at 4 wk
	PR: 50% decrease from baseline, confirmed at 4 wk	PR: 30% decrease from baseline, confirmed at 4 wk
	PD: 25% increase of one or more lesions, or appearance of new lesions	PD: 20% increase over smallest sum observed, or appearance of new lesions
	NC: neither PR or PD criteria met	SD: neither PR or PD criteria met
	2. Nonmeasurable disease	2. Nontarget lesions
	CR: disappearance of all known disease, confirmed at 4 wk	CR: disappearance of all target lesions and normalization of tumor markers, confirmed at 4 wk
	PR: estimated decrease of 50%, confirmed at 4 wk	
	PD: estimated increase of 25% in existent lesions or appearance of new lesions	PD: unequivocal progression of nontarget lesions, or appearance of new lesions
	Non-PD: persistence of one or more nontarget lesions and/or tumor markers above normal limits	NC: neither PR or PD criteria met
Overall response	1. Best response recorded in measurable disease	1. Best response recorded in measurable disease from treatment start to disease progression or recurrence
	2. NC in nonmeasurable lesions will reduce a CR in measurable lesions to an overall PR	2. Non-PD in nontarget lesion(s) will reduce a CR in target lesion(s) to an overall PR
	3. NC in nonmeasurable lesions will not reduce a PR in measurable lesions	3. Non-PD in nontarget lesion(s) will not reduce a PR in target lesion(s)
Duration of response	1. CR	1. Overall CR

From: date CR criteria first met	From: date of treatment start
To: date PD first noted	To: date PD first noted
2. Overall response	2. Overall response
From: date of treatment start	From: date CR or PR criteria first met (whichever status came first)
To: date PD first noted	To: date recurrent disease first noted
3. In patiënts who only achieve a PR, only the period of overall response should be recorded	3. SD
	From: date CR criteria first met
	To: date recurrent disease or PD first noted

* WHO = World Health Organization, RECIST = Response Evaluation Criteria in Solid Tumors, LD = longest diameter, CR = complete response, PR = partial response, PD = progressive disease, NC = no change, SD = stable disease.

Lesions that can only be measured unidimensionally are considered to be measurable (e.g., mediastinal adenopathy, malignant hepatomegaly).

Appendix B: Pre-procedural angiography

Indication:

Diagnostics prior to percutaneous chemosaturation in patients.

Aim:

Mapping vascular anatomy of the liver and embolization of extra hepatic branches, in preparing for chemosaturation treatment.

Contraindications:

- Acute life-threatening illness
- Liver failure or portal hypertension, with ascites, recent haemorrhage of oesophageal varices or encephalopathy.
- Liver cirrhosis Childs B or C
- Contraindications for chemotherapeutic agent (melphalan hydrochloride).
- Allergy or hypersensitivity for a component, material or drug used the Delcath Hepatic CHEMOSAT Delivery System, including heparin.
- Severe contrast allergy or contraindication for giving contrast (eGFR<30)
- Allergy for natural rubber latex (in the Delcath Hepatic CHEMOSAT Delivery System)
- History of congestive heart failure, with an LVEF < 40%
- Severe COPD or another chronic restrictive lung disease with FEV1 <30%
- Pregnancy or lactation
- Use of immunosuppressive medication or anticoagulants which cannot be stopped.

General information:

The pre-procedural angiography is made as a preparation to the chemosaturation treatment. Chemosaturation is used to treat patients with liver metastases, by percutaneous isolated hepatic perfusion in the angiography room. In preparation to this treatment, the first procedure will visualize the SMA (superior mesenteric artery), coeliac trunk, common hepatic artery and possible branches form the hepatic artery to other abdominal organs (gastroduodenal artery, right gastric

artery). Also an indirect portography will be performed by acquiring late imaging during arteriography). After delineation of the hepatic vascular anatomy, the risk of reflux of the chemotherapeutic agent to hepatico-enteric anastomoses such as the gastroduodenal or right gastric artery is determined. If there is a possible risk of reflux during the eventual treatment, these gastro-enteric anastomoses are embolised with coils or a vascular plug. Also arterial branches distal of the site of infusion with vascular supply to the stomach, pancreas, intestines and diaphragm (i.e. aberrant right gastric artery, aberrant left hepatic artery, aberrant phrenic arteries etc) are embolised.

Preparation:

Admission on the day of the procedure (arranged by requesting physician)
Check renal function and coagulation status when indicated
Fasting for eight hours for solid food and for two hours for fluids, before the procedure
Peripheral venous access (venflon 18 G of 20G)
Monitoring device for blood pressure, SpO2
Time-out procedure (see protocol)
Trash and laundry bags

General information:

See protocol: *Preventie van contrastmiddel-geïnduceerde nefropathie (CIN) bij volwassenen*.
See protocol: *Stollingsbeleid vastgesteld voor radiologisch onderzoek en behandeling*.
See protocol: *Contrastmiddel / Profylaxe bij ernstige contrastmiddelreacties Radiologie*.

Material:

Angioset
Injector with 150 cc contrast: Iomeron 300 mg/ml
3000 IE heparin
5 of 6 Fr sheath
Guidewired Terumo 0.035" 150 cm
Terumo Cobra 5 Fr 80 cm; Celiac 5 Fr 65 cm
Selective catheterization, dependent on anatomy; mostly with a High Flow Progreat microcatheter (Terumo)
Microcoils 0.018 Boston Scientific of Amplatzer 4 vascular plug

Procedure:

Approach via common LEFT femoral artery for hepatic arterial access and RIGHT femoral vein for retro-hepatic IVC access. Disinfect site of punction with red chlorhexidin and cover sterile. Anesthetize locally with lidocaine 1%, and place the 5 or 6 Fr sheath.
Selective catheterization of the SMA and hepatic artery, with a Terumo Cobra 5 Fr. Mapping the anatomy of the SMA, coeliac trunk and vascularization of the liver. Indirect portography via AMS. Coiling extra-hepatic branches with micro coils. After the procedure the sheath is removed and a vascular closure device is placed.

Images during the angiography:
Image of SMA including indirect portography

Max BV, 30 ml contrast, 6ml/sec, stijging 0.5, delay 0 sec. 1000PSI, lange serie van 50 sec, halverwege van 2 frames/sec naar 1 frame/sec.

Image of coeliac trunk

BV 31-38 bovenbuik, 20 ml contrast, 6ml/sec, stijging 0.5, delay 0 sec. 1000PSI, 2 frames/sec

Image of common hepatic artery

BV 31-38 bovenbuik, 15 ml contrast, 5ml/sec, stijging 0.5, delay 0 sec. 1000PSI, 2 frames/sec

Selective image with Progreat High Flow

BV 31-38 bovenbuik, 5-10 ml contrast, 1-3.5ml/sec, stijging 0.5, delay 0 sec. 750PSI, 2 frames/sec

XPerCT
Place the patient slightly off centre on the table (centre liver). XPerCT protocol. Abdomen Low. Delay 1-10sec, depending on staining of ‘region of interest’ on angiography. The injected volume is calculated using the formula “volume= (scan delay + scanning time) x injection rate”.

The injection rate can be deducted from the injection rate used during angiography. 250 PSI.

XPerCT Progreat selective
Center liver under flat panel detector. Protocol XPerCT Abdomen Low. Delay 1-10 sec dependant on the enhancement of the ‘region of interest’ at angiography. The injected volume is calculated using the formula “volume= (scan delay + scanning time) x injection rate”.

The injection rate can be deducted from the injection rate used during angiography. 250 PSI.

Aftercare and evaluation:
The intervention radiologist reports the results of the procedure in EZIS Radiology EPD and makes

a radiology report. After the procedure the patient returns to the ward. Nurses check the insertion area, blood pressure, temperature, ventilation, fluid balance and pain management.

Measure temperature before discharge

Fluid balance

- Peripheral venous access
- Make sure the patient has sufficient oral intake as dehydration increases the risk of contrast nephropathy
- Check urine production
    - Pain management
    - Additional medication can be prescribed when necessary, with approval of the attending physician
    - Patient can be discharged when pain is adequately managed and no other complications occurred.

Appendix C: Chemosaturation (PHP) treatment

Indication:

Treatment of patients with secondary tumors in the liver.

Aim:

Treatment of liver tumors with percutaneous infusion of the liver with chemotherapy followed by chemofiltration.

Contra-indications:

- Acute life-threatening illness
- Liver failure or portal hypertension, with ascites, recent haemorrhage of oesophageal varices or encephalopathy.
- Liver cirrhosis Childs B or C
- Contraindications for chemotherapeutic agent (melphalan hydrochloride).
- Allergy or hypersensitivity for a component, material or drug used the Delcath Hepatic CHEMOSAT Delivery System, including heparin.
- Severe contrast allergy or contraindication for giving contrast (eGFR<40)
- Allergy for natural rubber latex (the Delcath Hepatic CHEMOSAT Delivery System contains latex)
- History of congestive heart failure, with an LVEF < 40%
- Severe COPD or another chronic restrictive lung disease with FEV1 <30%
- Pregnancy or lactation
- Use of immunosuppressive medication or anticoagulants which cannot be stopped.

General information:

This treatment is meant to treat patients with liver tumors by percutaneous isolated perfusion of the liver with chemotherapy at the angiography room. Preferably this is done one week after the pre-procedural angiography. This treatment consists of catheterizing the proper hepatic artery and injecting the required amount of chemotherapeutic agent.

Preparation:

- Admission on the day of the procedure (arranged by requesting physician)

- Anti-emetic medication is started at the day of admission. First choice: metoclopramide 2-3 dd 10 mg i.v.
- On the day of treatment patients receive paracetamol 500 mg 3 times a day. If necessary morphinomimetics are added. When indicated, analgesia is continued for one week
- Prophylactic antibiotics are not indicated
- Check renal function and coagulation status when indicated
- Fasting for 8 hours for solid food and for two hours for fluids, before the procedure
- Peripheral venous access (venflon 18 G of 20G)
- A urinary catheter is inserted
- Monitoring device for blood pressure, SpO2
- Time-out procedure (see protocol)
- Trash and laundry bags

General information:
See protocol: *Preventie van contrastmiddel-geïnduceerde nefropathie (CIN) bij volwassenen*).
See protocol: *Stollingsbeleid vastgesteld voor radiologisch onderzoek en behandeling*).
See protocol: *Contrastmiddel / Profylaxe bij ernstige contrastmiddelreacties Radiologie*)

Material:
Angioset
Lidocaine 1%
Echo-cover set
Injector with 50 cc contrast; Contrastmiddel: Iomeron 300 mg/ml.
3000 IE heparin
5 of 6 Fr sheath
Guide wire Terumo 0.035" 150 cm
Terumo Cobra 5 Fr 80 cm; Celiac 5 Fr 65 cm
Selective catheterization, dependent on anatomy; mostly with a High Flow Progreat microcatheter (Terumo)
6F Angioseal or other closure device
Delcath Hepatic CHEMOSAT Delivery System

Procedure:
The procedure is performed under general anaesthesia. The patient has to be intubated with an

endotracheal tube and has to have a well working peripheral venous access. During the procedure the arterial pressure is to be measured constantly. Therefore an arterial line is inserted. A central venous triple-lumen line is placed to measure the central venous pressure and administering fluid and medication. Antibiotics are not necessary, unless a patient has a medical history of hepatobiliary surgery, which increases the risk of an infection. All vascular accesses will be made using ultrasound guidance and single anterior wall puncture entry.

A sterile punction of the right jugular vein is performed and the venous sheath of the Delcath Hepatic CHEMOSAT Delivery System is placed. Then the LEFT common femoral artery is punctured guided by ultrasound and a 5F/6F sheath is placed. After puncture and before introduction of the Delcath double balloon catheter heparin is injected (5000 IU). The Chemofuse Catheter is inserted via the femoral artery sheath and positioned in the proper hepatic artery. The tip of the catheter is placed distally of the gastroduodenal artery. The catheter is connected to an infusion pump with heparinized saline (1000 EH heparin/ 500cc saline). A puncture of the RIGHT common femoral vein follows and an 18F introducer sheath is placed (after sequentially dilatation with 8-14F dilatators). Through the 18F sheath the Isofuse® Isolation Aspiration Catheter is inserted and the tip is positioned at the level of the diaphragmatic hiatus. The ACT is checked and should be over 400 seconds. All catheters have now been inserted and the hemofiltration circuit is completed by connecting the different parts. The pump starts with 1000 rpms and is gradually increased to 2500 rpms, or to a speed at which the pressure of the hepatic vein is slightly negative. Typical flow speeds are 0.40 to 0.75 L/min; 0.80 L/min is the maximum speed.

The anaesthesiologist administers vasopressors that increase the MAP to above 65 mmHg. Then the double balloon catheter is inflated. When only the cranial balloon is inflated (the caudal balloon is empty), the balloon catheter is pulled back until the cranial balloon shuts off the junction of the inferior vena cava and the right atrium. Then the caudal balloon is inflated. Pump speed is decreased down to 1000 rpms. Isolation from the systemic circulation of the hepatic veins is checked by injecting contrast to assure there is no leakage. Now the pump speed is brought back to 2500 rpms. The flow in the hepatic arteries is checked by injecting contrast to check for spasm which, if encountered, is treated to resolution with intra-arterial nitroglycerin. Then the chemotherapeutics (melphalan hydrochloride) infusion starts: 3.0 mg/kg IBW (maximal total dose 220 mg) and is infused over 30 minutes with simultaneous filtration.

After a wash-out period of 30 minutes, the double balloon catheter is deflated. The Isofuse® Isolation Aspiration Catheter is flushed with saline. Then the Chemofuse and Isofuse® catheters are removed. The coagulation status is corrected with protamine sulphate based on the amount of heparin administered and the activated clotting time. Fresh frozen plasma, cryoprecipitate or fibrinogen replacement, and platelets are infused when needed to reverse coagulation.

Aftercare and evaluation:

After the procedure the patients in monitored at the Intensive Care Unit for at least 12 hours.

- Check haemostasis site of insertion
- Monitoring pulse, blood pressure, temperature, ventilation, fluid balance and pain scores
- Check coagulation (PT/INR, thrombocytes) and correction if necessary with co-fact, protamine and/or thrombocytes transfusion
- Anti-emetics if necessary, first choice: metoclopramid 2-3dd 10 mg i.v.
- Analgesia is necessary. Paracetamol 3dd 500 mg (if not sufficient, other medication can be prescribed)
- Additional medication can be prescribed when necessary, with approval of the attending physician

On day one, blood tests are performed (haemoglobin, leukocytes number and differentiation, PT/INR, thrombocytes, AST/ALT, Alkaline phosphatase, γGT, creatinine)

After 12 hours on the Intensive Care, the patient is transported to the normal ward, if the condition of the patient allows so. On the ward the following parameters are monitored:

- Check pulse and blood pressure three times a day
- Daily check of temperature
- Check fluid balance and pain management
- Anti-emetics if necessary, first choice: metoclopramide 2-3dd 10 mg
- Analgesia if necessary. Paracetamol 3dd 500 mg (if not sufficient, other medication can be prescribed)
- Additional medication can be prescribed when necessary, with approval of the attending physician
- After 2-3 days the patient is discharged, after a final blood test (haemoglobin, leukocytes number and differentiation, PT/INR, thrombocytes, AST/ALT, Alkaline phosphatase, γGT, creatinine).

Appendix D: General anaesthesiology techniques

Preparation: peripheral venous access, pulsoxymeter, blood pressure, 2 litre O2 via nasal tube

Medication schedule intravenous sedation and analgesia

Step 1 Bolus **0,05 mg Fentanyl** i.v.(Inject IV in 30 seconds)

Step 2 Flush with s 0.9% and check ventilation and saturation

Step 3 Bolus **1,0 mg (max 2,5 mg) Midazolam**, (consult radiologist)

Step 4 Flush with saline 0.9%

Step 5 Start procedure and titrate **0,5 mg (max 1,0 mg) Midazolam** if necessary, up to the desired level of sedation or analgesia.

Repeat step 4 and 5 until the desired level of consciousness. Wait several minutes to see the effect of midazolam, with a maximum dose of 7.5 mg (depending on the condition of the patient) .If necessary an extra 0,025 mg - 0,05 mg Fentanyl can be administred during the procedure, after 20-30 minutes, with a maximum dose of 0,1 mg.

Antidotum for midazolam

Step 1 Wake the patient and give a breathing command. **0,2 mg Anexate** i.v. in 15 sec.

If necessary use bag-valve-mask ventilation.

Step 2 Flush with saline 0.9%.

Step 3 Repeat every minute until the desired level of consciousness. Maximum dose is **1 mg**.

Step 4 If no effect: start BLS/ALS and turn on CPR signal (1000)

Antidotum for Fentanyl

Step 1 Wake the patient and give a breathing command. **0,2 mg Naloxon** i.v.

If necessary use bag-valve-mask ventilation.

Step 2 Repeat **0,2 mg Naloxon** i.v. if effect is insufficient, until the patient is breathing without support.

Step 3 Flush with saline 0.9% after every dosage.

Appendix E: Case report forms

Case Report Form

Percutane geïsoleerde leverperfusie voor de behandeling van irresectabele levermetastasen

**Informed Consent**		Informed Consent volledig ingevuld	ja	nee					

**Patient**		Studienummer							
		Geboortedatum							
		Initialen							
		Achternaam							
		WHO status	WHO1	WHO2	WHO3	WHO4			
		Gewicht (kg)							
		Lengte (cm)							
		Gebruik anticoagulantia							

**Inclusiecriteria**		Liver metastases of histologically confirmed primary colorectal adenocarcinoma	nee	ja					
		Resection of primary tumor > 1 month before IHP	nee	ja					
		Irresectable metastases confined to the liver based on CT-Thorax/abdomen and PET imaging	nee	ja					
		Metastases measurable on CT-scan	nee	ja					
		Life expectancy > 4 months	nee	ja					
		Leukocytes ≥ 3.0 X 10^9/L	nee	ja					
		Thrombocytes ≥ 100 X 10^9/L	nee	ja					
		Creatinine Clearance ≥ 40 ml/min	nee	ja					
		Bilirubin <17 µmol/L	nee	ja					
		ASAT							
		ALAT							
		gamma gt							
		alk fosf.							
		APTT < 32.5 sec	nee	ja					
		PT < 13.7 sec	nee	ja					

**Exclusiecriteria**		leeftijd (<18 en >65)	nee	ja					
		WHO performance ≥ 2	nee	ja					
		< 40% vitaal weefsel	nee	ja					
		aberrante vasculaire anatomie die IHP onmogelijk maakt	nee	ja					
		ernstige comorbiditeit	nee	ja					
		verstandelijk beperkt/gehandicapt	nee	ja					
		zwanger/inadequate anti-conceptie	nee	ja					

**Lab pre-op**		Hb		CC,C mmol/l					
		Ht		l/l					
		thrombocyten		CCC *10^9/l					
		leukocyten differentiatie		CC,C *10^9/l					
		ALAT		U/L					
		ASAT		U/L					
		bilirubine		CCC µmol/l					
		gamma GT							
		alkalisch fosfatase		U/L					
		LDH							
		creatinine klaring (cockroft)							
		glucose		mmol/ml					
		Natrium							
		Kalium							
		Fosfaat							
		albumine		CC g/l					
		amylase							
		aPTT							
		PT							
		INR		C,C					
		CEA		CCCC µg/l					
		PK-sample baseline	nee	ja					

Primaire tumor		datum ok								
		ziekenhuis								
		aard tumor	coloncar	melanoo	sarcoom	anders, namelijk....				
		lokalisatie colontumor	coecum	colon asc	flexura h	colon transversum				
			flexura li	colon des	sigmoid	recto-sig	rectum			
		Dukes stadium	A1	A2	B1	B2	C1	C2	D	?
		TNM	T	...	N	...	M	...		
		Pathologie	goed ged	matig ge	slecht ge	ongediff	onbekend			
		Chirurgie	hemicole	hemicole	transv re	sigmoid r	LAR	APR	anders,...	
		Radiotherapie start								
		Radiotherapie specificatie								
		Chemotherapie start								
		chemotherapie specificatie								

Beeldvorming (4 weke		CT abdomen-thorax	nee	ja					
		MRI abdomen-thorax	nee	ja					
		PET-scan	nee	ja					
		CT cerebrum	nee	ja					
		MRI cerebrum	nee	ja					
		metastasen anders dan in lever	nee	ja					

Levermetastasen		synchroon (aanwezig bij OK primaire darmtumor)								
		metachroon								
		diagnose op basis van	echo	CT	CT-angio	MRI	cytologie	histologie	per-op e	anders,...
		datum bovenstaand onderzoek								
		aantal metastasen								
		resterend gezond parenchym	<40%	≥40%						
		specificatie gezond parenchym								
		lokalisatie (segmenten 1 t/m 8)	....							

		Eerdere therapie levermetastasen						
		chirurgie	nee	ja, datum....				
		type chirurgie	thermoal	resectie, aantal segmenten....				
		chemotherapie eerstelijn	nee	ja, startd	einddatum.....			
		respons chemo eerste lijn	CR	PR	SD	PD	onbekend	
		chemotherapie tweede lijn	nee	ja, startd	einddatum.....			
		respons chemo tweede lijn	CR	PR	SD	PD	onbekend	

Angiografie		datum						
		nierfunctie						
		gebruik anticoagulantia						
		INR						
		risk of reflux?	nee	ja				
		vaten geëmboliseerd	nee	ja, namelijk...				

Leverperfusie		datum perfusie						
		perfusie doorgegaan	nee	ja				
		reden niet doorgegaan	1: extrah	2: <40%	3: anders, namelijk....			
		OK duur (min)	...	van...	tot...			
		tijd start infusie						
		PK sample 10 min na start melphalan infusie	nee	ja				
		bloedverlies (ml)						
		dosis melphalan (mg)						
		perfusievolume (ml)						
		lekkage (%)						
		tijd stop infusie						
		Pk sample 10 min na complete infusie	nee	ja				
		Pk sample 20 min na complete infusie	nee	ja				
		Pk sample 30 min na complete infusie	nee	ja				
		tijd start washout						
		tijd stop washout						
		Pk sample 5 min na washout	nee	ja				
		Pk sample 10 min na washout	nee	ja				

		Pk sample 15 min na washout	nee	ja				
		Pk sample 30 min na washout	nee	ja				
		hemostase insteek-opening liezen verlaten OK	nee	ja, zowel	afwijkend			
		complicaties tijdens operatie	nee	ja, toelichting:...				
		bijzonderheden	...					
		device failure?	nee	ja, toelichting:...				
		ICU voor 24 uur	nee	ja, toelichting:...				
		hemostase insteek-opening liezen op ICU	nee	ja, zowel	afwijkend			
		Pk sample 1 uur na washout	nee	ja				
		Pk sample 2 uur na washout	nee	ja				
		Pk sample 3,5 uur na washout	nee	ja				
		Pk sample 5 uur na washout	nee	ja				
		Pk sample 24 uur na washout	nee	ja				
Lab post-op		Hb		CC,C mmol/l				
		Ht		l/l				
		thrombocyten		CCC *10^9/l				
		leukocyten differentiatie		CC,C *10^9/l				
		ALAT		U/L				
		ASAT		U/L				
		bilirubine		CCC µmol/l				
		gamma GT						
		alkalisch fosfatase		U/L				
		LDH						
		creatinine		CCCC µmol/l				
		creatinine klaring (cockroft)						
		glucose		mmol/ml				
		Natrium						
		Kalium						
		Bicarbonaat						
		Fosfaat						
		albumine		CC g/l				
		amylase						
		aPTT						
		PT						
		INR		C,C				
		PK-sample baseline	nee	ja				
post-operatieve check		INR 1,2 ≥	nee	ja				
		aPTT in normal range	nee	ja				
		ASAT/ALAT normaal (in 10% van baseline)	nee	ja				
		Hb > 9 g/dL (met of zonder transfusie voor procedure)	nee	ja				
		thrombocyten > 50.000/mm3	nee	ja				
		analgesie met paracetamol genoeg	nee	ja				
		tijdens opname temp >38,5	nee	ja				
		hemostase liezen/hals	nee	ja				
		bloedtransfusie nodig gehad	nee	ja, ...	PC's			
		ontslagdatum	...					
Post-operatieve gegev		SAE	nee	ja, fax SAE formulier				
		Niet-hematologische toxiciteit	nee	ja, namelijk...				
		rapporteur	naam:....					

CRF Follow-up

		datum							
		follow-up visite no:							
beeldvorming follow-(		CT-scan thorax-abdomen datum							
		diameter tumor							
		RECIST criteria	CR	PR	SD	PD			
		....							
Lab follow-up		Hb				glucose			
		Ht				Natrium			
		thrombocyten				Kalium			
		leukocyten differentiatie				Fosfaat			
		ALAT				albumine			
		ASAT				amylase			
		bilirubine				aPTT			
		gamma GT				PT			
		alkalisch fosfatase				INR			
		LDH							
		creatinine							
		creatinine klaring (cockroft)							
		PK-sample	nee	ja					
follow-up		late complicaties	nee	ja, namelijk...					
		progressie	nee	ja, hepat	ja, extrahepatisch:...				
		progressie op basis van	CT-abdon	kliniek, namelijk...					
		QOL	....						
		rapporteur							

CRF niet regulier bezoek

		Reden	Klachten	Adverse event		
		Adverse event	SUSAR	SAE		
		Gemeld aan hoofdonderzoeker	Nee	Ja		
		Datum melding	Datum			
Onderzoek		Temperatuur (gemeten in oor, in Celcius)	Getal			
		Hemoglobine	Getal			
		Leukocyten	Getal			
		Trombocyten	Getal			
		Kreatinine	Getal			
		Bilirubine	Getal			
		Albumine	Getal			
		INR	Getal			
		CRP	Getal			
Beeldvorming		....	Nee	Ja		
		VOD	Nee	Ja		
		Abces	Nee	Ja,	....	
		Progressie ziekte	Nee	Ja		
		Heropname	Nee	Ja		
		Reïnterventie	Nee	Ja, chirur	Ja, radiol	Ja...

CRF einde studie

Reden		Exclusie (	Overlijde	Patient h	Device Malfunction	
Datum exit studie		Datum				
Is de patiënt behandeld met geïsoleerde leverperfusie?		Nee	Ja			

Appendix F: EORTC QLQ Questionnaires

EORTC QLQ-C30 (version 3)

Wij zijn geïnteresseerd in bepaalde dingen over u en uw gezondheid. Wilt u alle vragen zelf beantwoorden door het getal te omcirkelen dat het meest op u van toepassing is. Er zijn geen "juiste" of "onjuiste" antwoorden. De informatie die u geeft zal strikt vertrouwelijk worden behandeld.

Wilt u uw voorletters invullen: |_|_|_|_|
Uw geboortedatum (Dag, Maand, Jaar): |_|_|_|_|_|_|_|_|_|
De datum van vandaag (Dag, Maand, Jaar): 31|_|_|_|_|_|_|_|_|_|

	Helemaal niet	Een beetje	Nogal	Heel erg
1. Heeft u moeite met het doen van inspannende activiteiten zoals het dragen van een zware boodschappentas of een koffer?	1	2	3	4
2. Heeft u moeite met het maken van een <u>lange</u> wandeling?	1	2	3	4
3. Heeft u moeite met het maken van een <u>korte</u> wandeling buitenshuis?	1	2	3	4
4.. Moet u overdag in bed of in een stoel blijven?	1	2	3	4
5. Heeft u hulp nodig met eten, aankleden, u zelf wassen of naar het toilet gaan?	1	2	3	4

**Gedurende de afgelopen week:**	Helemaal niet	Een beetje	Nogal	Heel erg
6. Was u beperkt bij het doen van uw werk of andere dagelijkse bezigheden?	1	2	3	4
7. Was u beperkt in het uitoefenen van uw hobbies of bij andere bezigheden die u in uw vrije tijd doet?	1	2	3	4
8. Was u kortademig?	1	2	3	4
9. Heeft u pijn gehad?	1	2	3	4
10. Had u behoefte te rusten?	1	2	3	4
11. Heeft u moeite met slapen gehad?	1	2	3	4
12. Heeft u zich slap gevoeld?	1	2	3	4
13. Heeft u gebrek aan eetlust gehad?	1	2	3	4
14. Heeft u zich misselijk gevoeld?	1	2	3	4

<u>Wilt u a.u.b. naar de volgende bladzijde gaan</u>

**Gedurende de afgelopen week:**	**Helemaal niet**	**Een beetje**	**Nogal**	**Heel erg**
15. Heeft u overgegeven?	1	2	3	4
16. Had u last van obstipatie? (was u verstopt?)	1	2	3	4
17. Had u diarree?	1	2	3	4
18. Was u moe?	1	2	3	4
19. Heeft pijn u gehinderd in uw dagelijkse bezigheden?	1	2	3	4
20. Heeft u moeite gehad met het concentreren op dingen, zoals een krant lezen of televisie kijken?	1	2	3	4
21. Voelde u zich gespannen?	1	2	3	4
22. Maakte u zich zorgen?	1	2	3	4
23. Voelde u zich prikkelbaar?	1	2	3	4
24. Voelde u zich neerslachtig?	1	2	3	4
25. Heeft u moeite gehad met het herinneren van dingen?	1	2	3	4
26. Heeft uw lichamelijke toestand of medische behandeling uw <u>familieleven</u> in de weg gestaan?	1	2	3	4
27. Heeft uw lichamelijke toestand of medische behandeling u belemmerd in uw <u>sociale</u> bezigheden?	1	2	3	4
28. Heeft uw lichamelijke toestand of medische behandeling financiële moeilijkheden met zich meegebracht?	1	2	3	4

Wilt u voor de volgende vragen het getal tussen 1 en 7 omcirkelen dat het meest op u van toepassing is

29. Hoe zou u uw algehele <u>gezondheid</u> gedurende de afgelopen week beoordelen?

1	2	3	4	5	6	7
Erg slecht						Uitstekend

30. Hoe zou u uw algehele "<u>kwaliteit van het leven</u>" gedurende de afgelopen week beoordelen?

1	2	3	4	5	6	7
Erg slecht						Uitstekend

© Copyright 1995 EORTC Study Group on Quality of Life. Alle rechten voorbehouden. Version 3.0

EORTC QLQ – LMC21

Soms zeggen patiënten dat ze de volgende klachten of problemen hebben. Wilt u aangeven in welke mate u deze klachten of problemen gedurende de afgelopen week heeft ervaren door het getal te omcirkelen dat het meest op u van toepassing

Gedurende de afgelopen week:	Helemaal niet	Een beetje	Nogal	Heel erg
31. Heeft u moeite gehad met eten?	1	2	3	4
32. Had u het gevoel sneller dan normaal voldaan te zijn als u at?	1	2	3	4
33. Hebt u zich zorgen gemaakt over gewichtsverlies?	1	2	3	4
34. Had u problemen met uw smaakvermogen?	1	2	3	4
35. Had u een droge mond?	1	2	3	4
36. Had u een pijnlijke mond of tong?	1	2	3	4
37. Bent u minder actief geweest dan u had willen zijn?	1	2	3	4
38. Had u prikkelende handen of voeten?	1	2	3	4
39. Had u pijn in de maagstreek?	1	2	3	4
40. Had u last in de maagstreek?	1	2	3	4
41. Waren uw huid of ogen geel (geelzucht)?	1	2	3	4
42. Heeft u pijn in uw rug gehad?	1	2	3	4
43. Had u het gevoel dat u "trager" was dan anders?	1	2	3	4
44. Voelde u zich futloos?	1	2	3	4
45. Had u moeite met sociale contacten met vrienden?	1	2	3	4
46. Was het moeilijk om over uw gevoelens te praten met uw familie of vrienden?	1	2	3	4
47. Hebt u last gehad van stress?	1	2	3	4
48. Vond u het minder makkelijk om u te amuseren?	1	2	3	4
49. Maakte u zich zorgen over uw toekomstige gezondheidstoestand?	1	2	3	4
50. Maakte u zich zorgen over uw familie in de toekomst?	1	2	3	4
**De voorbije vier weken:**				
51. Heeft de ziekte of de behandeling ervan negatieve gevolgen gehad voor uw seksleven?	1	2	3	4

© QLQ-LMC21 Copyright 2002 EORTC Quality of life Group. Alle rechten voorbehouden

Appendix G: References

1. Manfredi S, Lepage C, Hatem C, Coatmeur O, Faivre J, Bouvier AM. Epidemiology and management of liver metastases from colorectal cancer. Annals of surgery 2006;244:254-9.

2. Vahrmeijer AL, van Dierendonck JH, Keizer HJ, et al. Increased local cytostatic drug exposure by isolated hepatic perfusion: a phase I clinical and pharmacologic evaluation of treatment with high dose melphalan in patients with colorectal cancer confined to the liver. British journal of cancer 2000;82:1539-46.

3. Pingpank JF, Libutti SK, Chang R, et al. Phase I study of hepatic arterial melphalan infusion and hepatic venous hemofiltration using percutaneously placed catheters in patients with unresectable hepatic malignancies. Journal of clinical oncology : official journal of the American Society of Clinical Oncology 2005;23:3465-74.

4. Miao N, Pingpank JF, Alexander HR, Steinberg SM, Beresneva T, Quezado ZM. Percutaneous hepatic perfusion in patients with metastatic liver cancer: anesthetic, hemodynamic, and metabolic considerations. Annals of surgical oncology 2008;15:815-23.

5. Rothbarth J, Pijl ME, Vahrmeijer AL, et al. Isolated hepatic perfusion with high-dose melphalan for the treatment of colorectal metastasis confined to the liver. The British journal of surgery 2003;90:1391-7.

6. van Iersel LB, Koopman M, van de Velde CJ, et al. Management of isolated nonresectable liver metastases in colorectal cancer patients: a case-control study of isolated hepatic perfusion with melphalan versus systemic chemotherapy. Annals of oncology : official journal of the European Society for Medical Oncology / ESMO 2010;21:1662-7.

7. Yoon DY, Park JH, Chung JW, Han JK, Han MC. Iatrogenic dissection of the celiac artery and its branches during transcatheter arterial embolization for hepatocellular carcinoma: outcome in 40 patients. Cardiovascular and interventional radiology 1995;18:16-9.

8. Sakamoto I, Aso N, Nagaoki K, et al. Complications associated with transcatheter arterial embolization for hepatic tumors. Radiographics : a review publication of the Radiological Society of North America, Inc 1998;18:605-19.

9. Dudeck O, Bulla K, Wieners G, et al. Embolization of the gastroduodenal artery before selective internal radiotherapy: a prospectively randomized trial comparing standard pushable coils with fibered interlock detachable coils. Cardiovascular and interventional radiology 2011;34:74-80.
