# Supplementary material for: Prospective evaluation of percutaneous hepatic perfusion with melphalan as a treatment for unresectable liver metastases from colorectal cancer
Source: PLoS One. 2022 Jan 13;17(1):e0261939. doi: 10.1371/journal.pone.0261939 (PMC8758076; doi:10.1371/journal.pone.0261939)
Supplement: Attachment — Submitted filename: Response to reviewers.docx [file pone.0261939.s003.docx]

**Respone to the reviewers**

Thank you for reviewing our manuscript. We have appreciated your comments and tried to incorporate them in our manuscript. Trying to keep things comprehensive, we responded to the academic editor and reviewer(s) in blue.

Academic editor:

This is a prospective study on a complex locoregional chemotherapy procedure named percutaneous hepatic perfusion. Percutaneous hepatic perfusion was performed with melphalan (high-dosage - approximately 200 mg) and hemofiltration.

The sample size is small (8 patients) and the primary endpoint is response. Considering the small sample size, it is not possible to investigate about the effect of selected variables on response (logistic regression analysis). Secondary endpoints include adverse events that resulted very limited (probably in relation to the use of hemofiltration and the skill of the Authors).

The scientific contribution is important but, in my opinion, the Authors should support with more emphasis the reasons to perform this locoregional chemotherapy procedure.

**Reply:** Although systemic therapy is currently considered the first-line treatment modality in patients with unresectable CRLM, it can be accompanied by serious side-effects. We hypothesised that M-PHP would be a good alternative with limited systemic side-effects for metastatic CRC patients with liver-only disease. It has already been demonstrated that M-PHP is a safe, well-tolerated procedure in patients with liver metastases from ocular melanoma (Cardiovasc Intervent Radiol. 2019;42(6):841–852). Unfortunately, the current study was terminated prematurely due to slow recruitment. Several factors such as the availability of alternative therapies (e.g. systemic chemotherapy or radioembolization) and competing trials with systemic drugs or intra-arterial therapies probably contributed to this slow recruitment. This, together with a tumor response rate and median OS that did not meet our expectations (unfavorable compared to the study by Rothbarth et al. in which patients with CRLM were treated with isolated hepatic perfusion using a high dose of melphalan (Br J Surg. 2003;90(11):1391–1397)) implies that there is currently no clear role for M-PHP in patients with CRLM outside of clinical trials.

The indications for M-PHP are still not defined in the international scientific literature.

International guidelines consider locoregional chemotherapy as third line for unresectable CRCLM.

The Authors protocol (as reported in the supporting information file), considers M-PHP for the CRCLM patients for whom “No other standard systemic treatment options (are) available”, but, at same time, include patients “Candidate for neoadjuvant chemotherapy as determined during the multidisciplinary meeting to down size the tumor.” and “Patients (they had) received no, one or more courses of systemic therapy”.

**Reply:** It is correct that relatively few exclusion criteria were incorporated in the study protocol whereby most patients with histologically proven, unresectable CRLM were indeed eligible for inclusion. As was shown in Fig. 1, in 4/16 patients that were discussed during a multidisciplinary team meeting prior to possible inclusion, were excluded from participation because first-line systemic therapy was preferred. Table 2 shows that all patients in the current study had received one or more types of systemic therapy before receiving M-PHP.

The ability to perform PHP may increase the role of hepatic perfusion for treatment of multiple tumor histologies since this procedure allows for sequential treatments to be delivered with lower morbidity. The management of unresectable liver metastases is a significant clinical problem that requires the combined efforts of multiple providers to develop an integrated approach for each patient. Continued evaluation of hepatic perfusion in these patients is necessary so that its role in this integrated approach can be more clearly defined.

***Minor changes***

Abstract page 3, line 6: please, added “with hemofiltration”.

**Reply:** We added these words.

Materials and Methods, Procedure details, page 7, line 12: please, clarify if the 16-F double-balloon catheter was positioned percutaneously or by surgical preparation of the femoral (or saphenous) vein.

**Reply:** We made alterations accordingly.

Table 4: please, precise if patients with grade 3/4 leukopenia/neutropenia received granulocyte colony-stimulating factors.

**Reply:** All patients received a subcutaneous injection of granulocyte-colony stimulating factor within 72h after each M-PHP procedure. We added a sentence in the caption of Table 4.

Table 4: please, re-format line 4 (gr).

**Reply:** We re-formatted Table 4.

In Discussion, the Authors should clarify with more details the reasons for believing and choosing a complex procedure such as M-PHP. In particular the Authors should clarify the reasons to propose percutaneous hepatic perfusion in CRCLM patients not previously submitted to systemic therapy or after failure of the first line.

**Reply:** See also one of the replies to the academic editor. We believe that there is currently no clear role for M-PHP in patients with CRLM outside of clinical trials. In an attempt to clarify this, some changes were made to the manuscript.

In Discussion, the Authors should elaborate on the reasons of the very limited adverse events.

**Reply:** In a pharmacological study that was conducted in 2017, de Leede et al. demonstrated that the mean extraction rate of the GEN 2 hemofiltration system is 86%. (Cardiovasc Intervent Radiol. 2017;40:1196–1205). This means that only a small fraction of melphalan that is administered in the hepatic artery will eventually reach the systemic circulation. Additionally, all patients receive a subcutaneous injection with granulocyte colony-stimulating factor after each M-PHP procedure in an attempt to limit any systemic side-effects as much as possible. We made some changes to the Discussion section.

In Discussion, as concerning the limitations paragraph, page 12, line 8, please added: “…definitive conclusions. In relation to this problem, the importance of multi-center recruitment, as performed in other locoregional chemotherapy trials […please cite this reference… Mambrini A, Sanguinetti F, Pacetti P, Caudana R, Iacono C, Guglielmi A, Guadagni S, Del Freo A, Fiorentini G, Cantore M. Intra-arterial infusion of 5-fluorouracil, leucovirin, epirubicin, and carboplatin (FLEC regimen) in unresectable pancreatic cancer: results of a ten-year experience. In vivo. 2006; 20: 751-6.], must be emphasized.

**Reply:** We made alterations accordingly.
