# Supplementary material for: Prospective evaluation of percutaneous hepatic perfusion with melphalan as a treatment for unresectable liver metastases from colorectal cancer
Source: PLoS One. 2022 Jan 13;17(1):e0261939. doi: 10.1371/journal.pone.0261939 (PMC8758076; doi:10.1371/journal.pone.0261939)
Supplement: Attachment — Submitted filename: Clementi Review.docx [file pone.0261939.s004.docx]

**Prospective evaluation of** **percutaneous hepatic perfusion with melphalan as a treatment for unresectable liver metastases from colorectal cancer.**

This is a prospective study on a complex locoregional chemotherapy procedure (melphalan percutaneous hepatic perfusion using hemofiltration).

The paper is very interesting but this is a very complex type of locoregional chemotherapy for hepatic metastases.

In 8 patients the procedure has been performed percutaneously. The sample size is too small to demonstrate that the introduction of a 16F catheter is always possible without complications in all types of femoral arteries (also arteries with a diameter lower than 7 mm).

Minor changes suggested:

The authors should elaborate the previous concepts in Material and Methods and in Discussion.
