# Supplementary material for: Prospective evaluation of percutaneous hepatic perfusion with melphalan as a treatment for unresectable liver metastases from colorectal cancer
Source: PLoS One. 2022 Jan 13;17(1):e0261939. doi: 10.1371/journal.pone.0261939 (PMC8758076; doi:10.1371/journal.pone.0261939)
Supplement: Attachment — Submitted filename: Response to reviewers - 2 docx.docx [file pone.0261939.s005.docx]

**Response to the reviewers**

Thank you for reviewing our manuscript. We have appreciated your comments and tried to incorporate them in our manuscript. Trying to keep things comprehensive, we responded to the academic editor and reviewer(s) in blue.

----------------------------------------------------------------------------------------------------------------

Reviewer #1: The manuscript entitled ‘Prospective evaluation of percutaneous hepatic perfusion with melphalan as a treatment for unresectable liver metastases from colorectal cancer’ with the aim to prospectively evaluate the efficacy and safety of M-PHP in patients with unresectable CRLM.

The manuscript can be further improved.

Sample size calculation
Page 9. the write up on the sample size requires further improvement. Information on margin of error, power of study to be stated. The statement of ‘16.5% around the observed proportion’ not clear.

Reply: Unfortunately, no standard power calculation was performed since no clear null-hypothesis could be formulated. We choose a sample size that allows the response percentage to be determined with sufficient accuracy, i.e. with a sufficiently narrow confidence interval. Assuming a true response percentage of 60%, a sample size of 34 patients will yield a two-sided confidence interval of length 0.33 (± 16.5% around the observed proportion). Adjustments were made to our manuscript to clarify this.

Page 10, what range refers to, min – max or IQR to be clearly stated.

Reply: We changed made alterations accordingly, e.g. see page 10: ‘Median melphalan dose was 220 mg (ranging from 190 to 220) for the first cycle and 220 mg (ranging from range 160 to 220) for the second cycle.’

All percentage figures to be at least 1 decimal point.
Reply: We made alterations accordingly. e.g. see page 10: ‘The one-, two-, and three-year OS was 50.0%, 50.0%, and 0% respectively.

For Figure 1, more information could be added such as intervention, assessment, outcome variables, duration/period etc.

Reply: Figure 1 is in our opinion to show the enrolment of patients, and why patients were not eligible for study inclusion. This is all provided in the figure. Elaborating on for instance intervention or outcome variables obtained distracts, in our opinion, from the information currently given in Figure 1.

For Table 2, 3, 4, dash (-) to be denoted in the table footnote.

Reply: Changes were made accordingly. See Table 2, 3 and 4.

For Table 3, days to be stated as the initial measurements which then converted to months. This needs to be stated in the methodology. mo to be denoted in table footnote.

Reply: Changes were made accordingly. See page 9: ‘Median OS, median PFS, and hPFS were measured in days and subsequently converted into months.’ Also changes were made accordingly in the footnote of Table 3.

In the Reference list, reference number 28 and 29 to conform with the journal format i.e journal name.

Reply: Changes were made accordingly. See reference 28 and 29

Reviewer #2: The paper is very interesting but this is a very complex type of locoregional chemotherapy for hepatic metastases.
In 8 patients the procedure has been performed percutaneously. The sample size is too small to demonstrate that the introduction of a 16F catheter is always possible without complications in all types of femoral arteries (also arteries with a diameter lower than 7 mm).
Minor changes suggested:
The authors should elaborate the previous concepts in Material and Methods and in Discussion.

Reply: This study aimed to evaluate the outcome in patients with colorectal liver metastases treated with percutaneous hepatic perfusion with Melphalan. The safety of this procedure has already been reported by Meijer et al. (Cardiovasc Intervent Radiol. 2019;42(6):841-852.) and is referred to in the last paragraph of the discussion. Until now, arterial access has not been a reason for exclusion of patients for this procedure in our hospital. Therefore, we do not elaborate extensively on this topic in the current paper.

Additional changes:

Figure one has been replace. The first box had a textual error, it said: ‘accessed for eligibility.’ It has now been changed to: assessed for eligibility.’
